# Genetic structure of drone congregation areas of Africanized honeybees in southern Brazil

**DOI:** 10.1590/S1415-47572009005000083

**Published:** 2009-12-01

**Authors:** Thais Collet, Alexandre Santos Cristino, Carlos Fernando Prada Quiroga, Ademilson Espencer Egea Soares, Marco Antônio Del Lama

**Affiliations:** 1 Departamento de Genética e Evolução, Universidade Federal de São Carlos, São Carlos, SPBrazil; 2 Departamento de Genética, Faculdade de Medicina de Ribeirão Preto, Universidade de São Paulo, Ribeirão Preto, SPBrazil

**Keywords:** drone congregation area, africanization, honeybee reproduction, population genetics

## Abstract

As yet, certain aspects of the Africanization process are not well understood, for example, the reproductive behavior of African and European honeybees and how the first Africanized swarms were formed and spread. Drone congregation areas (DCAs) are the ideal place to study honeybee reproduction under natural conditions since hundreds of drones from various colonies gather together in the same geographical area for mating. In the present study, we assessed the genetic structure of seven drone congregations and four commercial European-derived and Africanized apiaries in southern Brazil, employing seven microsatellite loci for this purpose. We also estimated the number of mother-colonies that drones of a specific DCA originated from. Pairwise comparison failed to reveal any population sub-structuring among the DCAs, thus indicating low mutual genetic differentiation. We also observed high genetic similarity between colonies of commercial apiaries and DCAs, besides a slight contribution from a European-derived apiary to a DCA formed nearby. Africanized DCAs seem to have a somewhat different genetic structure when compared to the European.

## Introduction

Since the introduction of the African honeybee subspecies *Apis mellifera scutellata* into Brazil in 1956 there has been a dramatic change in the genetic makeup of European-derived honeybee populations in South and Central America in less than 50 years (Schneider *et al.*, 2004). The replacement of the European by Africanized populations, and the strong predominance of African traits in the hybrid formed with the spread of African swarms throughout the New World, have been well documented (Lobo *et al.*, 1989; Smith *et al.*, 1989; Quezada-Euán and Paxton, 1999; Clarke *et al.*, 2001; Collet *et al.*, 2006).

Although several reasons for the prevalence of African traits in the resultant hybrid have been proposed (Schneider *et al.*, 2004), two important issues are, as yet, not well understood, namely, the reproductive behavior of African and European honeybees and how the first Africanized swarms were formed and spread. The reproductive behavior of *A. mellifera* under natural conditions can be assessed through studies on drone congregation areas (DCAs). These are where hundreds of drones from various colonies gather in the same geographical area for mating. The main properties of a DCA are: 1) the highly mixed drone population from many surrounding apiaries; 2) the formation of these areas takes place independent of the presence of queens; and 3) the clustering of flying drones can be found every year in the same place (Jean-Prost, 1958). The composition of drone congregations is of great consequence for the genetic structure of honeybee colonies, as the number of colonies represented in a DCA affects the genetic diversity of new colonies (Baudry *et al.*, 1998). Thus, DCAs are ideal for studying honeybee reproduction under natural conditions.

Most of the studies concerning DCA properties were hereto based on honeybee populations in Europe (Baudry *et al.*, 1998; Koeniger *et al.*, 2005a, 2005b; Kraus *et al.* 2005a), Asia (Punchihewa *et al.*, 1990; Koeniger *et al.*, 1994; Kraus *et al.*, 2005b; Wattanachaiyingcharoen *et al.*, 2008) or Africa (Free, 1987; Muerrle *et al.*, 2007). Few studies on Africanized DCAs have been undertaken, these being related to drone racial identification for European queen-mating inferences in African-derived honeybee areas (Loper and Fierro, 1991), to the identification of the degree of Africanization in certain areas (Eischen and Rubink, 1997), or yet to assess the seasonal abundance of European and African-derived drones in DCAs for areas undergoing Africanization (Quezada-Euán and May-Itzá, 2001). There are no studies concerning the genetic structure of DCAs in Africanized areas of South and Central America.

In southern Brazil, near the towns of Lajeado and Eldorado do Sul (Rio Grande do Sul State), seven DCAs, located close to commercial apiaries of European-derived or Africanized colonies, were identified. The presence of European-derived and Africanized colonies in the area presented an opportunity for checking how these bees cooperate in forming a DCA. In this study, and by means of seven microsatelite loci, we analyzed the genetic structure of seven drone congregations and four commercial European-derived and Africanized apiaries. Besides estimating the number of mother colonies drones originated from, we also analyzed whether those visiting the DCAs originated from one or more subpopulations. Herein, we will describe (1) the low genetic differentiation among seven DCAs, (2) the genetic similarity between each DCA and the surrounding apiaries, and (3) the low representation of the European mitotype in those DCAs close to European-derived colonies.

## Material and Methods

###  Sampling

**Figure 1:**
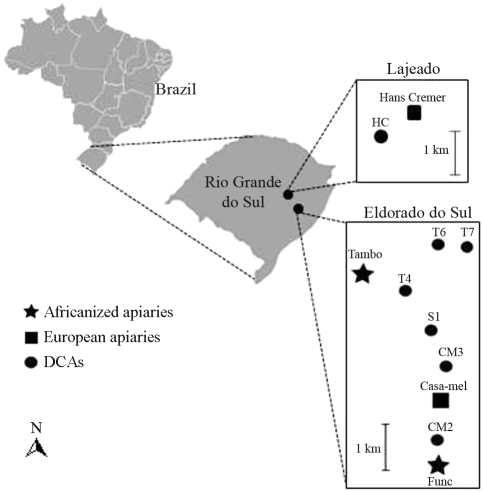
Location of the DCAs and apiaries sites in Lajeado and Eldorado do Sul (Rio Grande do Sul state).

A total of 410 *A. mellifera* drones were sampled from seven DCAs located near Africanized (T4, T6, T7 and S1) or European-derived apiaries (CM2, CM3, and HC) in Lajeado (30° 05' 44.1” S; 51° 40' 07.8” W) and Eldorado do Sul (29° 25' 43.1” S; 51° 58' 29.5” W) (Figure 1). All the DCAs were sampled in September, 2002, when the production of drones is high. Samples from CM2 were collected on two subsequent days and at different times, and were denominated CM2H1 (14:00-15:00 h) and CM2H2 (15:00-16:00 h). All drone congregations were very close, one to the other, except for DCA HC, which was located 76 km apart (Figure 1). Flying drones were captured with an aerial trap (Taylor, 1984) and kept at -20 °C until use. Workers were collected from four apiaries (Funcionários, Tambo, Casa-mel and Hans Cremer) located near these DCAs, so as to assess the effects of the presence of Africanized and European-derived colonies on DCA composition. The numbers of drones and workers per DCA and apiary are given in Table 1.

###  DNA extraction and analysis

Total DNA was extracted from the legs of the drones or from the thorax of a worker (one worker per colony) from each apiary by means of the phenol-chloroform method (Sheppard and McPheron, 1991).

Seven microsatellite loci (A14, A24, A43, A79, A88, A113 e B124) were amplified according to Estoup *et al.* (1995) and Solignac *et al.* (2003). Polymerase chain reaction (PCR) amplifications were performed in a 10 μL total volume containing 1 U of Platinum *Taq* DNA polymerase (Invitrogen), 10x *Taq* DNA polymerase buffer, 2.2 mM MgCl_2_ (except for A24, A43 and B124 with 2.5 mM MgCl_2_), 100 μM of each dNTP, 0.5 μM of each primer and 1 μL of template DNA for all primer pairs. The forward primer for each marker was labeled with one of three fluorescent dyes (Fan, Vic or Ned; Applied Biosystems). PCR conditions consisted of an initial 2 min denaturation step at 94 °C followed by 30 cycles of 94 °C for 30 s, annealing temperatures at 55 °C (A24, A43 and B124) or 62 °C (A14, A79, A88 e A113) for 30 s, and 72 °C for 30 s, plus a final extension at 72 °C for 10 min. The PCR products were run on a MegaBace 750-GE capillary sequencer using ET 550-R MegaBace (GE) as the internal size standard. MegaBace Fragment Profiler 1.2 software was used for allele identification.

The mtDNA pattern of the samples from the apiaries and DCAs was identified through *Dra*I restriction patterns of the COI-COII intergenic region for the former, and *Dra*I restriction patterns of the 16S fragment for the latter, respectively. The polymerase chain reaction (PCR) was carried out according to Garnery *et al.* (1992) for COI-COII, and digestion with *Dra*I enzyme to Collet *et al.* (2006). Amplification and restriction of the 16S fragment was undertaken according to Collet *et al.* (2007).

###  Data analysis

The number of alleles per locus (n) and expected heterozygozity (H_e_) were estimated with GENEPOP v3.4 software (Raymond and Rousset, 1995). We used either Fstat v2.9.3 software (Goudet, 2001) to calculate pairwise Fst values and their significant deviation from zero, as well as allelic richness (a_r_), or the sum of the number of alleles actually observed in each population when considering differences in sample size. In order to reveal whether there is genetic structure encompassing the data set, molecular variance analysis (AMOVA) was undertaken with Arlequin v2000 software (Schneider *et al.*, 2000). The same program was used to assess hierarchical multi-loci AMOVA. Variance was then partitioned into variance components and distributed among the groups (HC + T4, T6, T7, S1 + CM2, CM3), and among and within DCAs.

Colony v1.3 software (Wang, 2004) was used for estimating the number of colonies contributing drones to a given DCA. Five replicate analyses were performed with different seed numbers to calculate the number of colonies.

## Results

The number of alleles, allelic richness and genetic diversity of the DCAs and apiaries appear in Table 1. The number of alleles ranged from 5 (A24) to 17 (A14), with an average of 11.15 ± 2.2 alleles per locus for DCAs and 11.03 ± 2.4 for the apiaries. We found an average allelic richness of 7.9 ± 1.3 alleles per locus for the DCAs and 8.3 ± 1.5 for the apiaries.

Each drone sampled had its mtDNA origin assessed through *Dra*I restriction patterns of the 16S fragment. The DCA T7 and HC showed one and two drones, respectively, with a European pattern (*carnica/ligustica*) while all the remaining drones (n = 407) exhibited an African pattern. *Dra*I restriction patterns of the COI-COII mtDNA region permitted classifying the samples from apiaries located near the DCAs as either European (E), Africanized (A) or mixed (E/A) (Table 2).

No significant difference (Fst = 0.0025) was found between samples of the DCA CM2 collected during two consecutive days in two different periods. Due to the genetic homogeneity, CM2 was considered as a single unit for further analyses.

The DCA spatial pattern was reflected at the genetic level. Hierarchical AMOVA revealed that the greatest variation (99.9%) occurred among individuals within the DCAs themselves (Table 3). As a consequence, the six DCAs at Eldorado do Sul, closely distributed along ~5 km, did not show any population sub-structuring. The pairwise comparison also showed a low differentiation among most of the DCAs and apiaries with European-derived colonies (Figure 1 and Table 4). As expected, Hans Cremer and Casa-mel apiaries showed the greatest differentiation to the DCAs sampled (Table 4).

**Figure 2:**
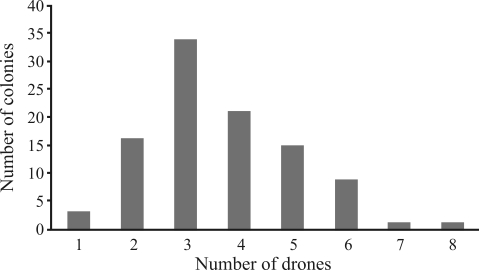
Number of colonies contributing drones to the DCAs.

Based on genotypic data, we estimated the number of colonies contributing drones to each DCA as: T4-17.8 ± 0.45; T6-26.2 ± 0.84; T7-26; S1-8; CM2-58 ± 1.23; CM3-13; HC - 21.2 ± 0.45. Since the F_st_ values revealed low levels of differentiation, we considered all DCAs as constituting a single unit, in order to estimate the number of drone-contributing colonies. The total number of colonies was 100.6 ± 1.34 (mean ± SD), thus proving that DCAs are constituted of poorly related drones of multi-colonial origin. The distribution of drones over the colonies followed a normal distribution (Wilks Shapiro Normality Test, W = 0.91; p > 0.1), ranging from one to eight contributed drones per colony, without any sign of skewed distribution or over-representation from any single colony (Figure 2).

## Discussion

Drones from the Africanized DCAs showed a higher number of alleles and gene diversity in comparison to a DCA from Germany (*A. m. carnica*) studied by Baudry *et al.* (1998) (Table 5). Higher gene variation, expressed as the expected heterozygosity, was found in the Africanized DCAs. African populations are significantly more variable than European, this having been interpreted as a consequence of the higher density of colonies and higher migratory ability of African subspecies when compared to the Europeans (Estoup *et al.*, 1995; Franck *et al.*, 1998). Moreover, Africanized honeybees are hybrid populations. Thus, a higher degree of genetic variation due to the contribution of some European and African alleles to the gene pool of the hybrid Africanized bee would be expected.

The most frequent alleles found in European populations (Estoup *et al.* 1995, Franck *et al.* 1998) are also the most frequent among Brazilian DCAs. This can be interpreted as either a significant European contribution to Africanized populations, or that these alleles are not efficient racial markers, since they are shared by the subspecies involved in the Africanization process. The latter interpretation seems to be more feasible, as allozymic (Lobo *et al.*, 1989; Del Lama *et al.*, 1990), mtDNA (Smith *et al.*, 1989; Clarke *et al.*, 2001; Collet *et al.*, 2006) and morphometric data (Diniz-Filho and Malaspina, 1995) all indicate a very high proportion of *A. m. scutellata* genes in Africanized honeybees.

No significant differences were found between samples of the DCA CM2 collected during two different periods. This finding is in accordance with previous observations that honeybees from European-derived or Africanized colonies do not present flight-activity at different periods in Neotropical regions (Cristino AS, unpublished results).

In Brazil, we found a higher variation in the number of drones contributed by a colony to a DCA, following a normal distribution. In contrast, most colonies in European DCAs (75%) contribute with the same number of drones, following a Poisson distribution (Baudry *et al.*, 1998). This differential contribution in Africanized colonies could be explained by differences in colony drone production due to genetic differences. The different ability for drone-production in the first African swarms would result in strong selection pressure, and therefore must have increased the differential drone contribution of African and European colonies to DCAs. Certainly, this was not the sole factor to determine the observed degree of mixture that tends towards African mitotypes. To address this question properly, it would be necessary to account for the additive genetic variability linked to drone production in Africanized colonies, this being an important trait to be worked by artificial selection.

Three out of the 410 drones analyzed showed the *carnica*/*ligustica* mtDNA pattern. The under-representation of European drones in a DCA was particularly evident in DCA HC, located 500 m from the Hans Cremer apiary (Figure 1 and Table 3), with colonies aggregating a large number of drones. This apiary was initially constituted of 40 *carnica* queens brought from Europe, the maternal lineage having been maintained through the production of new *carnica* queens. This result did not confirm the statement that drones choose the nearest DCA and that this choice boosts the genetic representation of local colonies (Koeniger *et al.*, 2005a).

The distribution of maternal nuclear alleles in each DCA and its closest apiary was not significantly different. These observations are consistent only if we consider that males copulating with a queen are a representative sample of the local bee population. The high number of colonies represented in a drone congregation makes mating between a queen and one of her brothers unlikely. If many colonies contribute equally to the DCA, a queen has an equal chance of insemination by a male from each colony within the recruitment area of a DCA. This results in low average relatedness between a queen's mates, thereby avoiding high inbreeding and homozigosis at the *csd* locus (Beye *et al.*, 2003; Beye, 2004) for her progeny, and consequently preventing the production of diploid drones, so avoiding a possible, heavy genetic load for the colony. Thus, high polyandry associated with the composition of drone congregations ensure very high intra-colony gene variation, thereby resulting in an efficient panmitic system.

The estimated number of colonies contributing with drones to the DCAs was much lower, when compared to the number presented by Baudry *et al.* (1998) for a *carnica* DCA. This result can be explained by the lower number of microsatellite loci used in this study, thereby decreasing the chances of detecting the number of families of brothers in each DCA. However, the most likely explanation for this result must be the distinct process by which DCAs are formed in Europe/USA and Brazil. Whereas DCAs are well defined and in small number in Europe and the USA, in Brazil they seem to be more continuously distributed. At least eight DCAs were identified in a study carried out in an area with a radius of three kilometers in Ribeirão Preto (São Paulo State, Brazil) (Martinez-Carantón OA and Soares AEE, unpublished results). Moreover, while in Europe the colonies are concentrated in apiaries and only a few feral swarms are found, in Brazil the number of feral swarms is high. Therefore, if a higher number of areas exist, one could expect a lower number of drones per DCA in Brazil and, as a consequence, a lower number of contributing colonies.

The major results of this study are the low genetic differentiation among the DCAs, the genetic similarity between workers of commercial apiaries and DCAs formed nearby, and the small contribution of a European apiary to a DCA formed nearby. The lack of genetic differentiation in the studied DCAs was also observed in samples of apiaries from 43 localities from northern to southern Brazil for twelve microsatellite loci (unpublished data). The homogeneous genetic composition of the DCAs showed on a micro-scale what happens on a macro-scale, *i.e.*, the homogeneous genetic conditions of Brazilian Africanized bee populations. Considering that 26 *scutellata* queens colonized much of the Americas and largely replaced European bees throughout their range in the New World, it is very impressive how this founder effect produced what has been considered one of the prime examples of a successful biological invasion known.

## Figures and Tables

**Table 1:** Sample size in each location (N), number of alleles (n), allelic richness (a_*r*_) and genetic diversity (Hd) by microsatellite loci in *Apis mellifera* drone congregation areas and apiaries from southern Brazil.

A24	T4	T6	T7	S1	CM3	HC	CM2H1	CM2H2	Hans Cremer	Funcionarios	Tambo	Casa-mel
N	29	60	63	10	18	42	42	48	22	20	34	16
n	7	7	7	5	6	7	7	7	6	6	6	6
a_*r*_	5.9	5.0	5.5	5.0	5.5	5.6	5.3	5.0	5.0	5.5	5.5	5.5
Hd	0.782	0.703	0.771	0.720	0.771	0.775	0.793	0.744	0.718	0.771	0.758	0.767

A43	T4	T6	T7	S1	CM3	HC	CM2H1	CM2H2	Hans Cremer	Funcionarios	Tambo	Casa-mel
N	29	60	62	10	19	42	42	48	22	20	31	14
n	10	14	13	7	12	13	16	15	13	11	10	11
a_*r*_	8.4	9.3	10.0	7.0	10.0	9.1	8.4	9.6	8.4	8.6	9.1	7.2
Hd	0.844	0.871	0.901	0.820	0.886	0.851	0.824	0.871	0.831	0.865	0.784	0.775

B124	T4	T6	T7	S1	CM3	HC	CM2H1	CM2H2	Hans Cremer	Funcionarios	Tambo	Casa-mel
N	29	60	61	10	19	41	41	48	22	18	33	13
n	11	13	11	8	10	14	15	13	13	12	12	11
a_*r*_	8.8	9.0	8.2	7.0	8.7	10.3	8.8	9.6	10.0	10.7	9.7	8.7
Hd	0.86	0.879	0.866	0.820	0.858	0.906	0.880	0.891	0.883	0.901	0.853	0.846

A14	T4	T6	T7	S1	CM3	HC	CM2H1	CM2H2	Hans Cremer	Funcionarios	Tambo	Casa-mel
N	30	59	59	10	20	46	44	48	24	21	34	18
n	11	16	17	6	11	14	17	15	14	14	14	15
a_*r*_	8.9	9.5	11.0	6.0	9.4	9.6	10.6	10.3	9.7	10.3	10.6	8.4
Hd	0.877	0.892	0.921	0.800	0.885	0.892	0.911	0.901	0.843	0.887	0.856	0.859

A79	T4	T6	T7	S1	CM3	HC	CM2H1	CM2H2	Hans Cremer	Funcionarios	Tambo	Casa-mel
N	30	58	59	10	20	45	44	47	24	21	36	19
n	10	15	13	7	7	13	14	14	14	12	11	14
a_*r*_	7.8	9.3	9.4	7.0	6.9	8.9	9.0	8.5	10.0	10.0	10.3	8.5
Hd	0.844	0.847	0.878	0.840	0.770	0.858	0.840	0.818	0.886	0.888	0.876	0.878

A88	T4	T6	T7	S1	CM3	HC	CM2H1	CM2H2	Hans Cremer	Funcionarios	Tambo	Casa-mel
N	29	58	56	10	20	46	44	48	24	20	31	18
n	8	12	10	4	5	9	12	11	9	11	13	9
a_*r*_	6.7	6.9	6.6	4.0	4.6	6.9	6.8	7.7	7.5	8.7	7.2	7.5
Hd	0.725	0.736	0.741	0.720	0.675	0.784	0.816	0.794	0.800	0.851	0.764	0.794

A113	T4	T6	T7	S1	CM3	HC	CM2H1	CM2H2	Hans Cremer	Funcionarios	Tambo	Casa-mel
N	30	59	57	10	20	46	44	48	24	21	36	19
n	11	11	12	6	9	9	13	11	12	12	8	10
a_*r*_	8.4	7.6	8.3	6.0	7.8	7.3	8.3	7.8	7.6	8.7	7.9	5.5
Hd	0.842	0.833	0.844	0.760	0.815	0.820	0.83	0.83	0.770	0.826	0.733	0.795

**Table 2:** Classification of the samples from apiaries located near the DCAs as European (E), Africanized (A) and both (E/A) according to COI-COII (*Dra*I) patterns.

	Haplotypes					
Apiary	A1	A4	A26	A28	C1	C2
Hans Cremer (E)					11	12
Funcionários (A)	6	13	1	1		
Tambo (A)	12					
Casa-mel (E/A)	3	14			2	

**Table 3:** Analysis of Molecular Variance (AMOVA) conducted for drone congregation areas of *Apis mellifera.* (df = degree of freedom; SS = sum of squares).

Source of Variation	df	SS	Variance components	% variation	p-value
Among groups	2	0.933	-0.00049	-0.10	> 0.05
Among DCAs within groups	6	3.124	0.00055	0.11	> 0.05
Within DCAs	401	199.621	0.49781	99.99	> 0.05

**Table 4:** Pairwise F_st_ values between pairs of *Apis mellifera* drone congregation areas.

	T4	T6	T7	S1	CM3	HC	CM2	H. Cremer	Func.	Tambo
T4										
T6	-0.0030									
T7	-0.0055	-0.0011								
S1	0.0007	-0.0112	-0.0084							
CM3	-0.0059	-0.0034	0.0018	-0.0044						
HC	-0.0009	0.0021	-0.0025	-0.0082	0.0022					
CM2	0.0064	0.0072	0.0079*	-0.0155	-0.0015	0.0040				

H. Cremer	0.0462*	0.0561*	0.0457*	0.0479	0.0488*	0.0310	0.0414*			
Func.	-0.0018	0.0033	-0.0026	-0.0022	0.0004	-0.0057	-0.0027	0.0352*		
Tambo	0.0019	-0.0013	0.0008	-0.0028	0.0038	-0.0107	-0.0142	0.0361	0.0040	
Casa-mel	0.0408*	0.0301*	0.0281*	0.0175	0.0292	0.0192	0.0319*	0.0594*	0.0227	0.0194

*p < 0.05.

**Table 5:** Comparison between Brazilian and European samples for the number of alleles (n_a_) and expected heterozygozity (*H*_e_). For the Brazilian samples, we present the average number of alleles and *H*_e_.

		Locus			
		A14	A79	A113	B124
Brazilian apiaries samples	n_a_	14.2 ± 0.4	12.7 ± 1.3	10.5 ± 1.7	12 ± 0.7
(n = 24)	*H*_e_	0.86 ± 0.01	0.88 ± 0.00	0.78 ± 0.03	0.87 ± 0.02

Brazilian DCAs samples	n_a_	14 ± 3.8	11.7 ± 2.8	10.5 ± 2.1	12.2 ± 2.2
(n = 44)	*H*_e_	0.88 ± 0.03	0.84 ± 0.03	0.82 ± 0.02	0.87 ± 0.02

European DCAs samples*	n_a_	12	11	9	12
(n = 142)	*H*_e_	0.47	0.84	0.59	0.73

*Data from Baudry *et al.* (1998).

## References

[Baudryetal1998] Baudry E., Solignac M., Garnery L., Gries M., Cornuet J.M., Koeniger N. (1998). Relatedness among honeybees (*Apis mellifera*) of a drone congregation. Proc R Soc Lond B.

[Beyeetal2003] Beye M., Hasselmann M., Fondrk M.K., Page R.E., Omholt S.W. (2003). The gene *csd* is the primary signal for sexual development in the honeybee and encodes an SR-type protein. Cell.

[Beye2004] Beye M. (2004). The dice of fate: The *csd* gene and how its allelic composition regulates sexual development in the honey bee, *Apis mellifera*. BioEssays.

[Clarkeetal2001] Clarke K.E., Oldroyd B.P., Javier J., Quezada-Euán G., Rinderer T.E. (2001). Origin of honeybees (*Apis mellifera* L. ) from the Yucatan peninsula inferred from mitochondrial DNA analysis. Mol Ecol.

[Colletetal2006] Collet T., Ferreira K.M., Arias M.C., Soares A.E.E., Del Lama M.A. (2006). Genetic structure of Africanized honeybee populations (*Apis mellifera* L. ) from Brazil and Uruguay viewed through mitochondrial DNA COI-COII patterns. Heredity.

[Colletetal2007] Collet T., Arias M.C., Del Lama M.A. (2007). 16S mtDNA variation in *Apis mellifera* detected by PCR-RFLP. Apidologie.

[DelLamaetal1990] Del Lama M.A., Lobo J.A., Soares A.E.E., Del Lama S.N. (1990). Genetic differentiation estimated by isozymic analysis of Africanized honeybee populations from Brazil and from Central America. Apidologie.

[Diniz-FilhoandMalaspina1995] Diniz-Filho J.A.F., Malaspina O. (1995). Evolution and population structure of Africanized honey bees in Brazil: Evidence from spatial analysis of morphometric data. Evolution.

[EischenandRubink1997] Eischen F.A., Rubink W.L. (1997). Using drone surveys to estimate Africanization levels. Am Bee J.

[Estoupetal1995] Estoup A., Garnery L., Solignac M., Cornuet J.M. (1995). Microsatellite variation in honeybee (*Apis mellifera* L. ) populations: Hierarchical genetic structure and test of the infinite allele and stepwise mutation models. Genetics.

[Francketal1998] Franck P., Garnery L., Solignac M., Cornuet J.M. (1998). The origin of West European subspecies of honeybees (*Apis mellifera*): New insights from microsatellite and mitochondrial data. Evolution.

[Free1987] Free J.B. (1987). Pheromones of Social Bees.

[Garneryetal1992] Garnery L., Cornuet J.M., Solignac M. (1992). Evolutionary history of the honey bee *Apis mellifera* inferred from mitochondrial DNA analysis. Mol Ecol.

[Jean-Prost1958] Jean-Prost P. (1958). Queen mating. Apimondia.

[Koenigeretal1994] Koeniger N., Koeniger G., Tingek S., Kelitu A., Mardan M. (1994). Drones of *Apis dorsata* (Fabricius 1793) congregate under the canopy of tall emergent trees in Borneo. Apidologie.

[Koenigeretal2005a] Koeniger N., Koeniger G., Pechhacker H. (2005a). The nearer the better? Drones (*Apis mellifera*) prefer nearer drone congregation areas. Insectes Soc.

[Koenigeretal2005b] Koeniger N., Koeniger G., Gries M., Tingek S. (2005b). Drone competition at drone congregation areas in four *Apis* species. Apidologie.

[Krausetal2005a] Kraus F.B., Koeniger N., Tingek S., Moritz R.F.A. (2005a). Using drones for estimating colony number by microsatellite DNA analyses of haploid males in *Apis*. Apidologie.

[Krausetal2005b] Kraus F.B., Koeniger N., Tingek S., Moritz R.F.A. (2005b). Temporal genetic structure of a drone congregation area of the giant Asian honeybee (*Apis dorsata*). Naturwissenschaften.

[Loboetal1989] Lobo J.A., Del Lama M.A., Mestriner M.A. (1989). Population differentiation and racial admixture in the Africanized honeybee (*Apis mellifera* L. ). Evolution.

[LoperandFierro1991] Loper G.M., Fierro M.M. (1991). Use of drone trapping and drone release to influence matings of European queens in an Africanized honey bee area (Hymenoptera, Apidae). J Apic Res.

[Muerrleetal2007] Muerrle T.M., Hepburn H.R., Radloff S.E. (2007). Experimental determination of drone congregation areas for *Apis mellifera capensis* Esch. J Apic Res.

[Punchihewaetal1990] Punchihewa R.W.K., Koeniger N., Koeniger G. (1990). Congregation of *Apis cerana indica* Fabricius 1798 in the canopy of trees in Sri Lanka. Apidologie.

[Quezada-EuanandMay-Itza2001] Quezada-Euán J.J.G., May-Itzá W.J. (2001). Partial seasonal isolation of African and European-derived *Apis mellifera* (Hymenoptera, Apidae) drones at congregation areas from subtropical Mexico. Ann Entomol Soc Am.

[Quezada-EuanandPaxton1999] Quezada-Euán J.J.G., Paxton R.J. (1999). Rapid intergenerational changes in morphology and behaviour in colonies of Africanized and European honey bees (*Apis mellifera*) from tropical Yucatan, Mexico. J Apic Res.

[RaymondandRousset1995] Raymond M., Rousset F. (1995). GENEPOP v. 1.2: Population genetics software for exact tests and ecumenicism. J Hered.

[Schneideretal2000] Schneider S., Roessli D., Excoffier L. (2000). Arlequin: A software for population genetics data analysis v. 2.000.

[Schneideretal2004] Schneider S.S., DeGrandi-Hoffman G., Smith D.R. (2004). The African honey bee: Factors contributing to a successful biological invasion. Annu Rev Entomol.

[SheppardandMcPheron1991] Sheppard W.S., McPheron B.A., Smith D.R. (1991). Ribosomal DNA diversity in Apidae. Diversity of the Genus *Apis*.

[Smithetal1989] Smith D.R., Taylor O.R., Brown W.M. (1989). Neotropical Africanized honey bees have African mitochondrial DNA. Nature.

[Solignacetal2003] Solignac M., Vautrin D., Loiseau A., Mougel F., Baudry E., Estoup A., Garnery L., Haberl M., Cornuet J.M. (2003). Five hundred and fifty microsatellites markers for the study of the honeybee (*Apis mellifera* L. ) genome. Mol Ecol Notes.

[Taylor1984] Taylor O.R. (1984). An aerial trap for collecting drone honeybees in congregation areas. J Apic Res.

[Wang2004] Wang J. (2004). Sibship reconstruction from genetic data with typing errors. Genetics.

[Wattanachaiyingcharoenetal2008] Wattanachaiyingcharoen W., Wongsiri S., Oldroyd B.P. (2008). Aggregations of unrelated *Apis florea* colonies. Apidologie.

